# Resvega, a Nutraceutical Preparation, Affects NFκB Pathway and Prolongs the Anti-VEGF Effect of Bevacizumab in Undifferentiated ARPE-19 Retina Cells

**DOI:** 10.3390/ijms231911704

**Published:** 2022-10-03

**Authors:** Randa Sghaier, Maude Perus, Clarisse Cornebise, Flavie Courtaut, Alessandra Scagliarini, Céline Olmiere, Virginie Aires, François Hermetet, Dominique Delmas

**Affiliations:** 1 UFR des Sciences de Santé, Université de Bourgogne, 21000 Dijon, France; 2 INSERM Research Center U1231—Cancer and Adaptive Immune Response Team, Bioactive Molecules and Health Research Group, 21000 Dijon, France; 3 Laboratoires Théa^®^, 12 Rue Louis-Blériot, 63000 Clermont-Ferrand, France; 4 Centre Anticancéreux Georges François Leclerc Center, 21000 Dijon, France

**Keywords:** AMD, angiogenesis, ocular diseases, anti-VEGF, Resvega, omega-3 fatty acids, resveratrol

## Abstract

Age-related macular degeneration (AMD) is an irreversible chronic degenerative pathology that affects the retina. Despite therapeutic advances thanks to the use of anti-vascular endothelial growth factor (VEGF) agents, resistance mechanisms have been found to accentuate the visual deficit. In the present study, we explored whether a nutraceutical formulation composed of omega-3 fatty acids and resveratrol, called Resvega^®^, was able to disrupt VEGF-A secretion in human ARPE-19 retina cells. We found that Resvega^®^ inhibits VEGF-A secretion through decreases in both the PI3K-AKT-mTOR and NFκB signaling pathways. In NFκB signaling pathways, Resvega^®^ inhibits the phosphorylation of the inhibitor of NFκB, IκB, which can bind NFκB dimers and sequester them in the cytoplasm. Thus, the NFκB subunits cannot migrate to the nucleus where they normally bind and stimulate the transcription of target genes such as VEGF-A. The IκB kinase complex (IKK) is also affected by Resvega^®^ since the nutraceutical formulation decreases both IKKα and IKKβ subunits and the IKKγ subunit which is required for the stimulation of IKK. Very interestingly, we highlight that Resvega^®^ could prolong the anti-angiogenic effect of Avastin^®^, which is an anti-VEGF agent typically used in clinical practice. Our results suggest that Resvega^®^ may have potential interest as nutritional supplementation against AMD.

## 1. Introduction

Age-related macular degeneration (AMD) is the eye disease most often responsible for severe vision loss in people older than 60. It is characterized by the progressive onset of neurodegeneration of the photoreceptor-retinal pigment epithelium (RPE) complex. RPE cells play a key role in the development of the different types of AMD. In fact, in age-related maculopathy, there is an accumulation of precursors, drusen, of variable shape, size and number, the most probable origin of which is the accumulation of residues of phagocytosis of the photoreceptors by cells of the RPE. This is then accompanied by an alteration of the RPE, with phenomena of hyper- or hypo-pigmentation [1]. In the atrophic or “dry” form, there is a progressive loss of RPE cells, due to the degeneration of the latter by apoptosis, which leads secondarily to the disappearance of the choriocapillaris, then of the photoreceptors in the macular region and finally, the severe loss of vision. Geographic atrophy occurs when the cell loss can no longer be compensated for by the spreading of the remaining cells that previously maintained the continuity of the epithelium [2]. Finally, in wet or exudative or even neovascular AMD (nAMD), the newly formed vessels are more fragile and lead to a diffusion of serum and blood, causing hemorrhages within the retina, which disrupts its organization, with, in particular, the appearance of exudates, intraretinal edema, scars or retinal detachments. In the absence of treatments, the exudates through the new choroidal vessels, cause the destruction of the photoreceptors [3]. All forms combined, it is the main cause of visual impairment in the elderly in industrialized countries, with more than 1 million affected individuals in France (approximately 8% of the population) and several hundred million worldwide [4]. There are generally three major clinical forms, but the term AMD corresponds to the most advanced stages of the pathology. So-called exudative or “wet” or nAMD, characterized by aberrant neoangiogenesis of the choroid and very rapid progression, affects only 10 to 15% of patients but accounts for 90% of severe vision loss [1,3]. The multiple molecular mechanisms underlying AMD are complex and still poorly understood. However, the major components involved in the pathogenesis of AMD are significant oxidative stress associated with the aging process, which appears to be the triggering factor for the pathology [5,6,7,8], and chronic inflammation eventually leading to the formation of the new choroidal vessels (choroidal neovascularization or CNV) observed in the most advanced and severe stages of the disease [9,10]. None of the clinical stages of AMD can be effectively prevented, treated or cured. Counseling patients on the importance of a healthy lifestyle including smoking cessation and a healthy diet is an important part of patient care. Since the 2000s, nAMD (the most severe form) has been treated with repeated intravitreal injections of antibodies (bevacizumab), humanized antibody fragment (ranibizumab), recombinant fusion protein (aflibercept) or, more recently, humanized single-chain antibody fragment (brolucizumab), directed against vascular endothelial growth factor (VEGF), the major angiogenesis-regulating factor (on average 7 injections per year) [11,12,13,14,15]. These targeted therapies have shown efficacy in stabilizing or even reversing the progression of the disease, but they do not constitute a definitive cure. They are indicated in the active phases of new vessel development but are not effective on healed or highly advanced forms, which implies that the diagnosis should be made in the early stages. In addition, recurrent fluid or exudation may persist despite anti-VEGF treatment. Thus, patients with refractory or recurrent AMD (approximately 1/3 of patients) may develop resistance mechanisms over the long term, leading to a reduction in therapeutic efficacy [16,17]. Moreover, the large number of repeated injections of anti-VEGF treatments required to maintain efficacy constitutes a heavy economic burden. It therefore seems urgent to develop new therapeutic strategies to block the neoangiogenic process, or to identify compounds capable of increasing the effectiveness of anti-VEGFs or making it possible to space out the injections.

Faced with this major public health problem, numerous studies and pharmaceutical companies have attempted to develop other VEGF inhibitors, which is the key factor in controlling neoangiogenesis, analogous to what is well known in models of tumor angiogenesis. In this field, we have highlighted the potential interest of a nutraceutical, Resvega^®^ (RGA), containing both resveratrol (RSV) and ω-3 fatty acids (eicosapentaenoic acid (EPA), docosahexaenoic acid (DHA)). This new formulation, based on the Age-Related Eye Disease Study 1 (AREDS-1) recommendations concerning the use of ω-3 fatty acids, was able to counteract angiogenesis in a preclinical mouse model of CNV [18]. Indeed, the combination of RSV and ω-3 fatty acids was able to decrease laser-induced CNV in mice, and a proteomic analysis of retinas revealed that mice supplemented with RGA present a specific enrichment in negative regulation of epithelial cell migration clusters in the retinas, including negative vasculature development, blood vessel development, angiogenesis, blood vessel morphogenesis and negative regulation of epithelial cell proliferation [18]. Very interestingly, the observed benefit relative to angiogenesis development produced by the omega-3 (ω-3) fatty acids/RSV combination was better than ω-3 fatty acids or RSV alone. A potential molecular explanation was recently suggested by some authors showing that RGA could reduce inflammation [19] and oxidative stress [20], and, on the contrary, induce autophagy [21] in retinal cell models. Very recently, we have demonstrated that the ω-3 fatty acids/RSV combination interferes with the transcriptional regulation of genes coding both for VEGF-R2 and VEGF-A [22]. Nevertheless, the molecular mechanisms by which RGA prevents angiogenesis in retina cells is still unclear.

In this novel study, we show the ability of an ω-3 fatty acids/RSV combination to counteract VEGF-A production over time due to an alteration of the AKT/PI3K and NFκB pathways. More specifically, RGA reduces the inhibitor of the κB (IκB) kinase (IKK) regulator complex that controls the future of NFκB, and the subunits P65 and P50 are diminished in nuclear fractions of the ARPE-19 retina cells treated with the nutraceutical formulation. Disruption of these two important angiogenesis signaling pathways increases the sensitivity of retina cells to anti-VEGF treatment. Pretreatment with RGA helps to prolong the treatment effect of Avastin^®^, an anti-VEGF commonly used in clinics to treat AMD patients. This finding provides evidence that this ω-3 fatty acids/RSV formulation could be used as a new therapeutic strategy in combination or not with anti-VEGF treatments in AMD therapy.

## 2. Results

### 2.1. RGA Inhibits VEGF-A Secretion over Time in Human ARPE-19 Retina Cells

**Figure 1:**
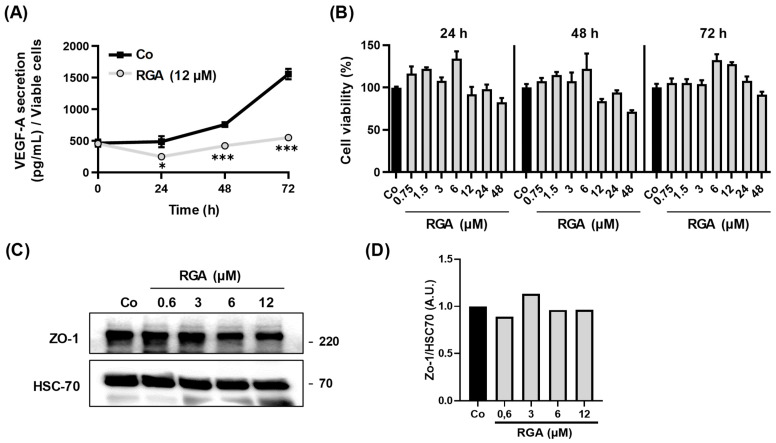
RGA formulation disrupts VEGF-A secretion over time in human ARPE-19 retina cells. (**A**) ARPE-19 retina cells were treated for 24, 48 and 72 h without serum with vehicle as control (Co) or with RGA (12 µM). VEGF-A secretion was measured in cell medium by ELISA assay. The data are mean ± S.D. of four independent experiments with n = 10. *p* values were determined by one-way ANOVA followed by Tukey’s multiple comparison test of treatments vs. control. * *p* < 0.05, and *** *p* < 0.001. (**B**) ARPE-19 cells were treated with 12 µM of RGA at 37 °C for 24, 48 and 72 h. The percentage of cell viability was determined by crystal violet assay. Results are expressed as a percentage of control (mean ± SD of three independent experiments with n = 10). (**C**) Immunoblot analysis of ZO-1 in RGA treated ARPE-19 cells with increasing concentration (0.6, 3, 6, 12 µM) or with vehicle (Co) for 24 h. HSC-70 was used as a loading control. (**D**) Densitometry quantification of western blotting. Data are expressed as the mean fold induction compared to control (Co).

One of the pro-angiogenic factors playing a major role in the development, progression and complications of AMD is vascular endothelial growth factor A (VEGF-A). VEGF-A is strongly involved in the abnormal growth of blood vessels and the increase in vascular permeability that leads to loss of vision [23]. We have previously been able to show that RGA supplementation was able to decrease VEGF secretion in an in vivo mouse model [18]. In undifferentiated human ARPE-19 retinal cells mimicking the cells affected by AMD [24], this nutraceutical formulation was also able to reduce the secretion of VEGF by approximately 20% after 24 h of treatment, without affecting cell viability [22]. Firstly, in this same human retinal cell model that is widely used to test the efficacy of anti-angiogenic compounds, we tested whether the anti-angiogenic effect of RGA lasted over time, increased or, on the contrary, decreased. For these experiments, ARPE-19 cells were treated with RGA over 72 h and, interestingly, ω-3 fatty acids/RSV formulation at 12 µM reduced VEGF-A secretion over time, as shown by the ELISA method (Figure 1). This reduction was accentuated from 48 h of treatment to reach a maximum difference at 72 h of treatment, where the formulation inhibited VEGF secretion by 64.6% compared to the control (Figure 1A). It is important to note that RGA (12 µM) has no significant impact on the cellular viability of human ARPE-19 retinal cells as a function of incubation time (kinetics observed between 0 h and 72 h) (Figure 1B).

Furthermore, the integrity of human retinal–endothelial barrier (REB) is maintained by tight junctions of the RPE cells in particular, which create a very tight monolayer, so that macromolecules cannot easily penetrate between the cells forming the retinal unit [25]. These tight junction proteins such as zonula occludens-1 (ZO-1) are essential to maintain retinal homeostasis and to conserve choroid microenvironment. Thereby, the loss of tight junctions, connected to the actin cytoskeleton through ZO-1, leads to retinal-barrier disruption, which is associated with the AMD-pathogenicity [25,26]. To explore the role of RGA in human RPE barrier integrity and permeability, we investigate its impact on the expression of the essential tight junctions’ proteins. Immunoblotting analysis revealed that RGA maintains ZO-1 expression in ARPE-19 cells.

### 2.2. RGA Disrupts AKT/mTOR Signaling Pathway

**Figure 2:**
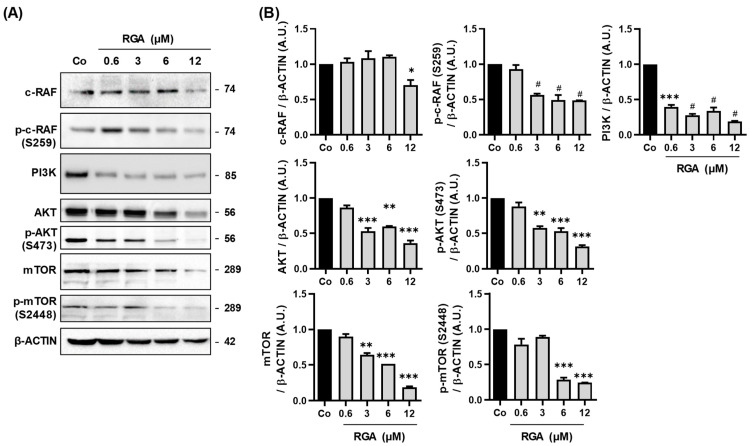
RSV/ω-3 fatty acids combination disrupts PI3K-AKT-mTOR signaling pathway. (**A**) Immunoblot analysis of c-RAF, phospho-c-RAF (p-RAF), PI3K, AKT, phospho-AKT (p-AKT), mTOR, phospho-mTOR (p-mTOR) in RGA-treated ARPE-19 cells with increasing concentration (0.6, 3, 6, 12 µM) or with vehicle (Co) for 24 h. β-ACTIN was used as a loading control. (**B**) Densitometry quantification of western blotting. Data are expressed as the mean fold induction ± SEM of three independent experiments. *p*-values were determined by a one-way ANOVA followed by Tukey’s multiple comparison test of treatments vs. control. * *p* < 0.05, ** *p* < 0.01, *** *p* < 0.001, and # *p* < 0.0001.

We next explored the mechanisms underlying the inhibitory effect of RGA on VEGF-A production. The very fine regulation of angiogenesis involves numerous pathways following activation by VEGF-A of its main receptor, VEGF-R2. Once activated, it in turn triggers many intracellular signaling pathways. These include, in particular, the ERK1/2 pathway on which we have shown that RGA has an effect [22], but also other pathways, for instance the PI3K-AKT-mTOR pathway, which is crucial for cell survival, regulation of vasomotion and angiogenesis [27,28] In order to understand the impact of RGA on VEGF-A pathway through PI3K-AKT-mTOR, we investigated its effect on the expression of key proteins involved in the signaling cascade. Immunoblotting analysis revealed that RGA strongly decreases phosphorylation of c-RAF, in a concentration-dependent manner, as well as the protein expression of PI3K, AKT, mTOR and their respective phosphorylated forms (Figure 2A,B).

### 2.3. RGA Inhibits NFκB Subunits and Its Kinase Inhibitor IκB

**Figure 3:**
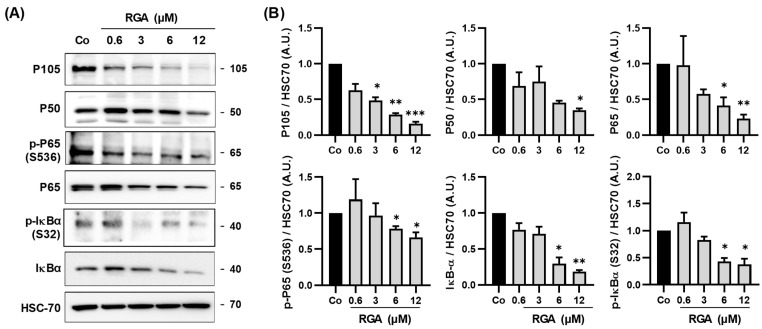
RSV/ω-3 fatty acids combination decreases NFκB complex protein expression. (**A**) Immunoblot analysis of P105, P50, phospho-P65, (p-P65), P65, phospho-IκBα (p-IκBα), IκBα in RGA-treated ARPE-19 cells with increasing concentration (0.6, 3, 6, 12 µM) or with vehicle (Co) for 24 h. HSC-70 was used as a loading control. (**B**) Densitometry quantification of western blotting. Data are expressed as the mean fold induction ± SEM of three independent experiments. *p* values were determined by a one-way ANOVA followed by Tukey’s multiple comparison test of treatments vs. control. * *p* < 0.05, ** *p* < 0.01 and *** *p* < 0.001.

It is well established that AKT regulates the phosphorylation of many proteins downstream of the PI3K-AKT-mTOR signaling pathway, such as the NFκB transcriptional complex, which plays an essential role in the transcriptional activation of the gene encoding the VEGF-A protein in human retina cells. This nuclear transcriptional factor is a multimeric complex constituted of the isoform P65 and P50 which is formed from a cytosolic precursor P105 protein. In response to multiple stimuli such as oxidative stress, reactive oxygen species (ROS) or inflammatory cytokines, the NFκB inhibitory factor, IκB, is rapidly phosphorylated, which results in its degradation and allows the activation of NFκB. This allows the release of NFκB dimer (P50/P65) and its translocation into the nucleus, where it binds to promoters of various target genes. In order to analyze the potential effect of RGA on these factors, ARPE-19 cells were treated with increasing concentrations of RGA. Immunoblotting analyses revealed that RGA decreases both P50 and its precursor P105 by around 66% and 84%, respectively, at 12 µM of RGA compared to the control (Figure 3A,B). Similarly, RGA diminished in a concentration-dependent manner the expression of P65 and its phosphorylated form by 77% and 34%, respectively, compared to the control. Interestingly, RGA strongly inhibited both IκB and its phosphorylation, suggesting a potential repercussion on the cellular distribution of NFκB subunits (Figure 3A,B).

### 2.4. RGA Inhibits Nuclear Relocalization of Dimer P65/P50 Subunits of NFκB

**Figure 4:**
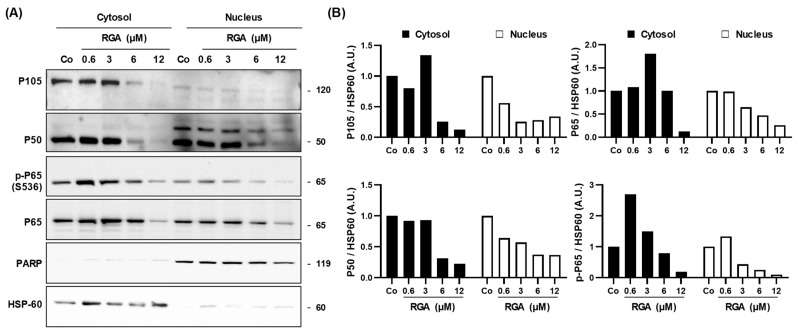
RGA disturbs the translocation of the P65 and P50 dimer subunits of NFkB into the nucleus of ARPE-19 cells. (**A**). Representative blot of P105, P50, P65 protein expression in concentration range of RGA (0.6, 3, 6, 12 µM)-treated or not (Co) ARPE-19 cells for 24 h. Nuclear fractions and cytosolic fractions are shown from three independent experiments. (**B**) Densitometry quantification of western blotting from the representative nuclear and cytosolic immunoblotting. Data are expressed as the mean fold induction.

In the processes of angiogenesis observed in AMD, the canonical NFκB activation pathway mainly applies to the dimers formed by the P65 and P50 subunits. Under normal conditions, the P65/P50 dimers are sequestered in the cytoplasm in an inactive form by their association with the inhibitory IκB proteins (inhibitor of NFkB). NFκB activation in response to a stimulus leads to the phosphorylation of IκB, and results in IκB ubiquitination and degradation. As a consequence, free P65/P50 dimers translocate into the nucleus to activate the transcription of specific target genes such as the gene coding for VEGF-A. Because RGA strongly inhibited the phosphorylation of IκB (Figure 3), this action could lead to a cytosolic sequestration of the P65/P50 subunits, thus preventing the dimer from playing its role as a transcriptional factor on the gene coding for VEGF-A in the nucleus of retinal cells. To assess the ability of RGA to prevent the relocalization of nuclear factors into the nucleus, we performed immunoblotting from a nucleus/cytoplasm extraction where poly (ADP-ribose) polymerase-1 (PARP-1) and heat shock cognate (HSC-70) proteins were used as controls for the nuclear and cytosolic fractions, respectively (Figure 4). While P50 was indeed expressed in the nuclear fraction in the control (Co), RGA decreased, in a concentration-dependent manner, the amount of this protein in the nuclear fractions compared with the control, reaching around 36.7% at 12µM of RGA (Figure 4A,B). The precursor of P50, P105, is more expressed in the cytosolic fraction, which is its normal localization, compared to the nuclear fraction. In both cytosolic and nuclear fractions, RGA treatment strongly decreased its protein expression by 12.6% and 34.0%, respectively, at 12 µM of RGA (Figure 4A,B). Similar to P50, the subunit P65 and its phosphorylated form (p-P65) were also strongly diminished in nuclear fractions compared with the control, by 26.3% and 9.8%, respectively, at 12 µM of RGA (Figure 4A,B).

### 2.5. RGA Inhibits Subunits of IκB Kinase

**Figure 5:**
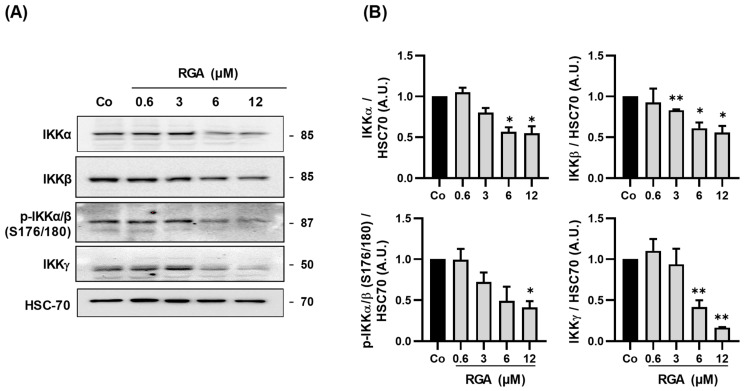
The RSV/ω-3 fatty acids combination decreases subunits of IκB complex protein expression. (**A**) Immunoblot analysis of IKKα, IKKβ, phospho-IKKαβ (p-IKKαβ), IKKγ in RGA-treated ARPE-19 cells with increasing concentrations (0.6, 3, 6, 12 µM) or with vehicle (Co) for 24 h. HSC-70 was used as a loading control. (**B**) Densitometry quantification of western blotting. Data are expressed as the mean fold induction ± SEM of three independent experiments. *p* values were determined by a one-way ANOVA followed by Tukey’s multiple comparison test of treatments vs. control. * *p* < 0.05 and ** *p* < 0.01.

Sequestration into the nucleus of NFκB subunits is under the double control of a multimolecular complex called IκB kinase (IKK). Indeed, this heterotrimercomplex kinase IKK controls the phosphorylation of inhibitors of κB (IκB) [29,30]. The two homologous kinases, IKKα and IKKβ subunits (85 and 87 kDa, respectively), form the IKK complex with the unrelated regulatory 52-kDa subunit IKKγ [31], also known as NEMO (NF-κB essential modulator) [32]. This complex is responsible for the phosphorylation of IκBα, which leads to its degradation and the release of the transcription factor NF-κB. RGA disrupts both the IκB phosphorylation (Figure 3) and diminishes the nuclear translocation of p65 and p50 and their phosphorylated forms, respectively (Figure 4). We then sought to determine whether the effects induced by RGA after 24 h of treatment were associated with a modulation of the key proteins constituting the IKK complex, which control the fate of NFκB. Immunoblotting revealed that RGA strongly decreases, in a concentration-dependent manner, the two subunit kinases IKKα and IKKβ and the phosphorylated form of the homodimer phospho-IKKαβ (p-IKKαβ). Inhibition was significant in all cases at 12 µM (Figure 5A,B). Very interestingly, IKKγ, which is required for the stimulation of IKK by upstream signals, was also strongly decreased by the concentration range of RGA (Figure 5A,B).

Altogether, the first data set demonstrated that RGA formulation is able to affect the canonical NFκB activation pathway by decreasing both the protein expression of P65/P50 dimers and their phosphorylated forms, but also to affect key regulators controlling their cellular distribution. This effect, which is induced by the RGA, then makes it possible to reduce the translocation of P65 and P50 in the nucleus and thus to prevent their role as nuclear transcriptional factors.

### 2.6. RGA Sensitizes AREP-19 Retina Cells to the Anti-Angiogenic Effect of Bevacizumab

**Figure 6:**
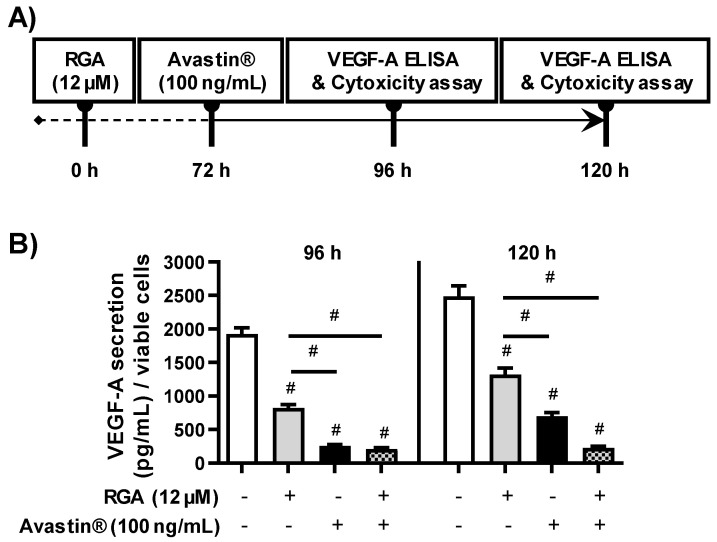
RSV/ω-3 fatty acids prolong the VEGF-A inhibition induced by Avastin^®^. (**A**) Experimental workflow. After 24 h of culture, ARPE-19 retina cells were treated with 12 µM of RGA or with vehicle during 72 h, and then exposed to Avastin^®^ at 100 ng/mL during 24 or 48 h. At each time point, the level of VEGF-A secreted was measured by ELISA assay as well as the percentage of viable cells by crystal violet assay. (**B**) Histograms of the concentration of secreted VEGF-A (pg/mL) normalized to cell viability after 24 or 48 h or treatment with Avastin^®^ (100 ng/mL) in ARPE-19 retina cells, and pretreated or not with RGA (12 µM). Data are expressed as the mean fold induction ± SEM of the three independent experiments. *p* values were determined by a one-way ANOVA followed by Tukey’s multiple comparison test to 2 groups (control group vs. treatment group and treatment group vs. treatment group). # *p* < 0.0001.

Because RGA induces VEGF-A secretion inhibition, at least involving the NFκB and PI3K-AKT-mTOR signaling pathways, the ω-3 fatty acids/RSV combination could sensitize human ARPE-19 retina cells to the anti-angiogenic effect of an anti-VEGF such as bevacizumab (Avastin^®^). We therefore examined whether RGA at 12 µM could delay the VEGF-A inhibitory effect of Avastin^®^ on ARPE-19. To explore this hypothesis, we pretreated ARPE-19 for 72 h at 12 µM and then exposed retina cells for 24 h to Avastin^®^ at 100 ng/mL before assessment of VEGF-A secretion at 96 and 120 h, which is the retinal cell culture time limit in our experiments (Figure 6A). Levels of VEGF-A secreted by ARPE-19 cells shown that RGA decreases VEGF-A from 58% and Avastin^®^ from 87% and the combination of RGA with Avastin^®^ is similar to Avastin^®^ alone (Figure 6B). But very interestingly, when the VEGF-A was measured 24 h later, the level had risen by around 15% with Avastin^®^ alone, whereas, in the cells pretreated with RGA, the anti-angiogenic effect of Avastin^®^ prolonged the inhibition at a level comparable to that observed 24 h earlier i.e., 91% of inhibition (Figure 6B).

## 3. Discussion

**Figure 7:**
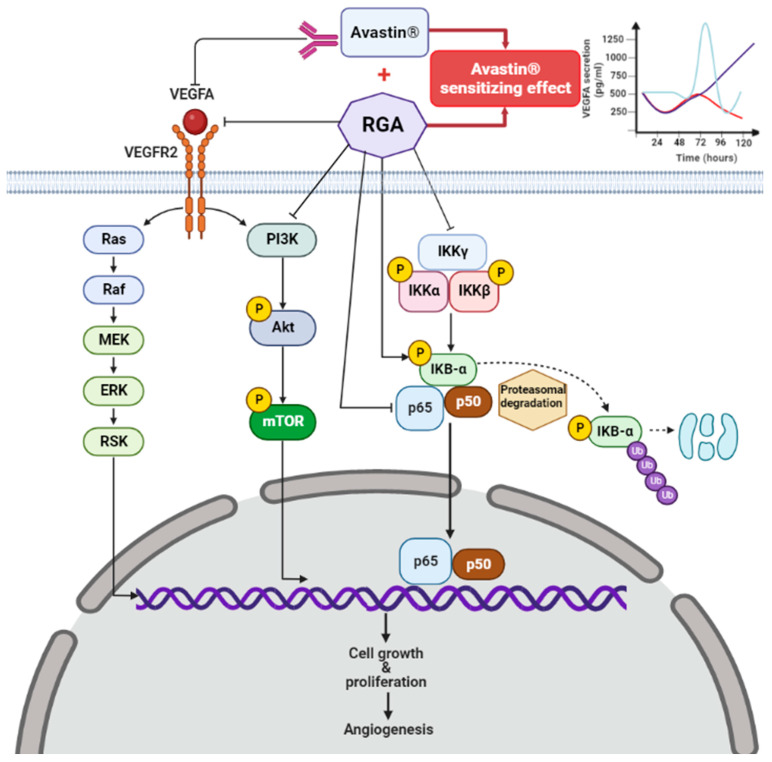
Schematic illustration of the novel proposed mechanisms of the anti-angiogenic impact of Resvega^®^ (RGA), which is able to target several angiogenesis molecular pathways in human ARPE-19 retina cell model. RGA counteracts angiogenic VEGF-A production through an alteration of the AKT/PI3K and NFκB pathways. In detail, RGA reduces the regulator complex IKK controlling the downstream NFκB subunits P65 and P50 belonging and their nuclear/cytosolic localization. This new RGA-targeted pathway complements data on the impact of RGA, which we have previously demonstrated to be involved in preventing activation of the MAPK signaling pathway in ARP19 cells [22]. The disruption of these two important angiogenesis signaling pathways likely leads to a sensitization of retina cells to the anti-VEGF treatment, resulting in an increased ability of Avastin^®^ to decrease VEGF-A secretion over time.

Age-related macular degeneration (AMD) is a disabling degenerative disease that reduces visual acuity in patients after the age of 50. Despite the development of new therapies to counteract neoangiogenesis through the development of anti-VEGF antibodies or agents (i.e., ranibizumab, aflibercept, bevacizumab, etc.) [33], treatment failure still occurs due to side effects and resistance. Herein, for the first time, through the use of a potential anti-angiogenic nutraceutical combining ω-3 fatty acids and RSV (Resvega^®^; RGA), we provide a mechanism by which RGA counteracts VEGF-A secretion through a modulation of the PI3K-AKT-mTOR pathway, disturbing the canonical pathway of NFκB and its regular IKK complex. These new insights complement the full spectrum of action of RGA on signaling pathways, which we and others have recently begun to describe. We previously demonstrated that RGA, as anti-VEGF formulation, prevents activation of the MAPK signaling pathway in ARP19 cells [18,22]. In total, the RGA-mediated disruption of these two important angiogenesis signaling pathways (i.e., PI3K-AKT-mTOR and MAPK signaling pathways) likely lead a prolongation of anti-angiogenic properties of bevacizumab (Avastin^®^), an anti-VEGF often used to treat AMD (Figure 7).

The phosphorylation of AKT leads to the expression of inflammatory cytokines (IL-6, IL-8, IL-18), which support the inflammatory environment involved in AMD pathogenesis, and contributes to VEGF secretion by the inflammatory-induced angiogenesis process [34]. Previous study has revealed that RGA, as an anti-inflammatory formulation, could modulate the expression of inflammatory cytokines such as IL-6 and IL-8 [20]. Our results suggest that RGA-mediated decreases in AKT phosphorylation may underlie the anti-inflammatory ability of RGA. If we determine here for the first time, to our knowledge, the ability of a nutraceutical containing both RSV and ω-3 fatty acids to increase ability of Avastin^®^ to decrease VEGF-A secretion over time, other studies will be necessary to mechanistically explore the impact of RGA/Avastin^®^ in ARPE 19 and to expand the use of RGA in combination with others anti-VEGF agents such as ranibizumab, aflibercept or brolucizumab.

AMD, which is a chronic, progressive and disabling retinal degenerative disease is characterized by two evolutionary forms: an exudative form, also called the “wet form”, and a dry form. In the exudative forms, nutritional supplementation can be associated with drug treatments. Indeed, the results of the Age-Related Eye Disease Study 1 (AREDS-1), a multicenter, randomized controlled clinical trial, showed the benefit of using large doses of micronutrients in the early stages of the disease [35]. The importance of doses of micronutrients, well beyond dietary intake, and the prospect of a therapeutic effect have illustrated a novel concept in ophthalmology that appeared at the beginning of the 2000s, that of health food. Various nutraceutical combinations have been developed, for example Longevinex^®^ which is a polyphenol combination including RSV and quercetin. A clinical trial in elderly patients in the USA showed that an oral administration of this polyphenol combination reduced neovascularisation, and the authors saw objective retinal and visual restoration similar to anti-VEGF therapy [36,37,38]. The drug treatments inhibiting the VEGF pathway are only effective in progressive exudative forms (wet forms, representing only 20% of clinical forms of AMD) and they are administered intravitreally. Their contraindications, useful clinical effects and undesirable effects stem from the route of administration and the effects of inhibiting VEGF (risk of infection, hemorrhage, etc.). Moreover, retinal cells are able to put resistance systems into place in order to produce VEGF-A regardless of the anti-VGF used. Indeed, various signaling pathways are induced to produce more VEGF-A, amplifying its secretion. Among these cellular pathways, the PI3K-AKT-mTOR pathway seems to be a target that could be used to disturb VEGF-A production. Various studies have shown in in vivo tumor models, that targeting this signaling pathway reduces angiogenesis and microvascular density [39,40]. In a similar manner, in the eye, inhibition of this pathway has been described as a new strategy to manage neovascularization in a variety of ocular diseases [41]. For example, recent studies have shown that the use of a selective inhibitor of class I PI3K and mTOR pathways blocks vascular leakage and reduces CNV lesion size in a laser-induced model of neovascular AMD [42]. Therefore, a combination of anti-VEGF with a nutraceutical such as RGA resulting from ω-3 fatty acids and RSV could act on the PI3K-AKT-mTOR pathway. The combination of the two therapies would thus aim to enhance the disease-modifying properties of the treatment of wet AMD. We show in the present study that RGA is able to inhibit the key protein of this signaling pathway by reducing the phosphorylation of mTOR as well as AKT and the PI3K protein (Figure 2). Subsequent to this inhibition, the NFκB pathway was also affected. RGA was therefore able to act both on the NFκB complex by reducing the protein expression of each subunit (P50 and P65) and on the IκB protein, which is a key protein in the NFκB complex (Figure 3). By inhibiting the phosphorylation of the NFκB complex, IκB can bind NFκB dimers and sequester them in the cytoplasm (Figure 3 and Figure 4). As a result, the NFκB subunits cannot migrate to the nucleus where they normally bind to and stimulate the transcription of target genes such as VEGF-A. Interestingly, another checkpoint controlling activation of NFκB, the IKK complex, is also affected by RGA, since the ω-3 fatty acids/RSV combination decreases both two subunits of IκB complex protein expression (IKKα and IKKβ) and the subunit IKKγ which is required for the stimulation of IKK (Figure 5). The decreases in VEGF-A secretion and the PI3K-AKT-mTOR-NFκB pathway by RGA reinforce the results of a recent laser-induced CNV in mouse model study in which supplementation for 14 days with RGA significantly reduced CNV development [18]. These in vivo results were also supported by a global proteome analysis on lasered retinas where we identified a specific enrichment in negative regulation of epithelial cell migration cluster in the retinas from mice supplemented with RGA, including negative vasculature development, negative blood vessel development, negative angiogenesis and blood vessel morphogenesis and negative regulation of epithelial cell proliferation [18]. It is worth noting that the pharmacokinetics of RGA demonstrated that after 21 days of RGA supplementation in mice, both RSV aglycons, but mainly RSV metabolites (especially RSV-3-O-sulfate, RSV-4′-O glucuronide and 3-O-glucuronide), were found in the retina and in the posterior pole [18].

Thus, by targeting relevant pathways, the ω-3 fatty acids/RSV combination could enhance or prolong the effect of anti-VEGF-A. To support this hypothesis, a pretreatment of retina cells with RGA followed by a treatment with 100 ng/mL of bevacizumab (Avastin^®^) made it possible to lengthen the inhibitory effect of Avastin^®^ on VEGF-A production (Figure 6). These important preliminary results, obtained using a combination of a nutraceutical and an anti-VEGF agent on a human retina cell line, pave the way for future studies because it may reduce the undesirable effects of anti-VEGF antibodies and prolong their effect. Furthermore, a recent study was able to show that RSV alone was able to reverse the adverse effect of bevacizumab in ARPE-19 retinal cell [43]. Studies are ongoing to determine whether a combination of ω-3 fatty acids and RSV could also reverse this side effect and enhance the effect of bevacizumab in in vivo models.

## 4. Materials and Methods

### 4.1. Cell Culture and Treatment

Human retinal pigmented epithelial cell line (ARPE-19) were obtained from the American Type Culture Collection (ATCC, Manassas, VA, USA). ARPE-19 were cultured in Dulbecco’s modified Eagle’s F12 medium (DMEM/F12) supplemented with 10% fetal bovine serum (FBS; Dutscher, Brumath, France), 1% penicillin/streptomycin at 37 °C in a 5% CO_2_ incubator and passaged twice per week or before they reach 80–85% confluency. These undifferentiated ARPE-19 cells spontaneously produce human RPE cell lines with normal karyology, which form polarized epithelial monolayers [44]. The cells were seeded for the different tests, at a constant density of 17,000 cells/cm^2^, to obtain identical experimental conditions.

After 24 h of cell culture, the culture medium was removed and cells were washed twice with Hank’s Balanced Salt Solution (HBSS, Dutscher, Brumath, France) and then incubated in DMEM/F12 with 1% FBS and 1% penicillin/streptomycin (Unit/mL) for 24 h. The cells were further incubated with Dimethyl Sulfoxide (DMSO), used as control, or increasing concentration of Resvega^®^ (RGA, Laboratoires Théa^®^, Clermont-Ferrand, France) (0, 6, 3, 6 and 12 µM) for 24 h. RGA is a nutraceutical formulation composed of Vitamin C 240 mg; Vitamin E 30 mg; Zinc 12.5 mg; Cu 1 mg; EPA 380 mg; DHA 190 mg; Lutein 10 mg; Zeaxanthin 2 mg; trans-RSV 30 mg).

Avastin^®^ (also called Bevacizumab; 25 mg/mL) was provided by Roche and used at a concentration of 100 ng/mL (dilution in DMEM/F12 medium with 1% FBS and 1% penicillin/streptomycin for treatment of the cells for 24 h and 48 h, with or without 72 h RGA pretreatment.

### 4.2. ELISA

The cells were seeded in a 96-well plate (5600 cells/well) 24 h before being treated or not in a final volume of 100 µL of cell culture media per well. Cell culture supernatants were assayed by ELISA for human VEGF-A (BMS277-2 Invitrogen, Waltham, MA, USA), according to the manufacturer’s protocol. Briefly, 50 µL of supernatant of each condition were diluted with 50 µL of sample diluent, loaded onto each well of 96 well plates coated with anti-human VEGF-A antibody, and finally incubated at room temperature on a microplate shaker for 2 h. After washing, 100 μL of biotin conjugated anti-human VEGF-A with horseradish peroxidase (HRP) was added and incubated for 1 h at room temperature, followed by 100 μL of substrate incubation. After 15 min, the absorbance was then measured at 450 nm using a Biochrom Assay UVM340 microplate reader.

### 4.3. Antibodies

For Western-Blot analyses, the following antibodies were used: c-RAF Rabbit pAb #9422 (1:1000); Phospho-c-RAF (Ser259) Rabbit pAb #9421(1:100); PI3K Rabbit pAb #4292 (1:1000); AKT Rabbit pAb#9272 (1:1000); Phospho-AKT (Ser473) Rabbit pAb #9271 (1:1000); mTOR (L27D4) Mouse mAb #4517 (1:1000); Phospho-mTOR (Ser2448) Rabbit pAb#2971(1:1000); β-ACTIN Mouse mAb#A1978(1:10,000); NF-κB1 p105/p50 (D4P4D) Rabbit mAb #13586 (1:1000); NF-κB p65 (D14E12) XP^®^ Rabbit mAb #8242 (1:1000); Phospho-NF-κB p65 (Ser536) (93H1) Rabbit mAb #3033 (1:1000); IκBα (L35A5) Mouse mAb #4814 (1:1000); Phospho-IκBα (Ser32) (14D4) Rabbit mAb #2859 (1:1000); IKKα Rabbit pAb #2682 (1:1000); IKKβ (D30C6) Rabbit mAb #8943(1:1000); IKKγ (DA10-12) Mouse mAb #2695 (1:1000); Phospho-IKKα/β (Ser176/180) (16A6) Rabbit mAb #2697 (1:1000); ZO-1 Rabbit pAb #13663 (1:1000). For ARPE-19 subcellular fractionation approach, the purity of cytoplasmic and nuclear fractions was determined with HSP60 (D6F1) XP^®^ Rabbit mAb #12165 (1:1000) and poly (ADP-ribose) polymerase-1 (PARP-1) (PARP) (Rabbit mAb# 9542 (1:1000), respectively. All antibodies were purchased from Cell signaling (Ozyme, Saint-Cyr-l’École, France) excluding 71 kDa heat shock cognate (HSC-70) Mouse mAb# sc7298 (1:10,000), which was purchased from Santa Cruz Biotechnology (Nanterre, France).

### 4.4. Western Blot Analysis

ARPE-19 cells were collected, washed with cold 1X phosphate-buffered saline (PBS Dutshcher, brumath France) centrifugate (5 min, 300× *g*, 4 °C), and then incubated for 30 min on ice with RIPA buffer (Radioimmunoprecipitation Assay buffer, 50 mM Tris-HCl, 150 mM sodium chloride, 0.5% sodium deoxycholate, 1% NP40 and 0.1% sodium dodecyl sulfate, pH 8) containing a protease inhibitor cocktail (Roche, Boulogne-Billancourt, France) a phosphatase inhibitor, sodium fluoride (50 mM) and a protease inhibitor, phenylmethylsulfonyl fluoride (PMSF; 100 µM, Sigma-Aldrich, St. Quentin Fallavier, France). After centrifugation (20 min, 16,000× *g*, 4 °C), the cell debris were eliminated and the protein concentrations were assessed using QuantiPro TM BCA kit (Bicinchoninic Acid; Sigma Aldrich, St. Louis, MO, USA). Samples were adjusted into denaturing loading buffer (50 mM Tris-HCl, 10% glycerol, 5% 2-mercaptoethanol, 2% sodium dodecyl sulfate, pH 6.8 and 0.1% bromophenol blue) and then denatured for 5 min at 95 °C prior to separation. Twenty micrograms of protein were separated on a 10% SDS-PAGE gel, and then transferred onto a nitrocellulose membrane. Nonspecific binding sites were blocked by incubation, for 1 h at room temperature, with 5% non-fat milk powder in PBST (PBS, 0.1% Tween 20, pH 7.2) before overnight incubation at 4 °C with specific primary antibodies which are diluted at 1:1000 in 5% milk-PBST or 5% BSA-PBST. Then, membranes were washed three times for 10 min, with PBST and subsequently incubated at room temperature for 1 h with appropriate horseradish peroxidase (HRP)-conjugated secondary antibodies (Jackson ImmunoResearch, Interchim, Montlucon, France) followed by exposure to enhanced chemiluminescence detection to ECL (Bio-Rad, Marnes-la-Coquette, France). Digital chemiluminescence images were acquired with a ChemiDocTM XRS+ imaging system (Biorad, Marnes-la-coquette, France). Blot analysis and/or band intensity determination was carried out with Image LabTM Software 6.0.1 (Biorad, Marnes-la-coquette, France).

### 4.5. Nuclear and Cytoplasmic Fraction Isolation

The cells were lysed in buffer (NE-PER; Pierce Biotechnology, Rockford, IL, USA), supplemented with a protease inhibitor cocktail (Halt; Pierce Biotechnology, Rockford, IL, USA), according to the manufacturer’s protocol. Protein concentrations were determined (BCA Kit; Pierce Biotechnology) before being loaded onto polyacrylamide gels.

### 4.6. Densitometry and Statistical Significance

The densitometry of blots was performed with ImageJ software (National Institutes of Health). Unless indicated in the legends of figures, the reported values represent the means of triplicate from one representative experiment repeated three times +/− SD. Statistical analysis was carried out with Prism GraphPad6.0 Prism software (GraphPad Software, La Jolla, San Diego, CA, USA). Data are shown as means ± standard deviation (SD) for triplicate assay samples of three independent experiments. The difference between mean values was determined by a one-way ANOVA followed by Tukey’s test for multiple comparisons. *p*-values < 0.05 were considered significant (* *p* < 0.05, ** *p* < 0.01, *** *p* < 0.001 and # *p* < 0.0001).

## 5. Conclusions

In this study, we showed that a nutraceutical enriched with ω-3 fatty acids and resveratrol, a polyphenol, could decrease VEGF-A secretion in retinal cells through a disruption of the PI3K-AKT-mTOR signaling axis and NFκB pathways. These first results were associated with a prolongation of the anti-angiogenic effect of Avastin^®^, an anti-VEGF usually used in clinical practice to treat AMD. Nonetheless, further investigations are needed to demonstrate the potential use of Resvega^®^ as nutritional supplementation with anti-VEGF antibodies against AMD, especially in preclinical models.

## Data Availability

The authors declare that all data supporting the findings of this study are available within the article.
